# Characterizing seasonal changes in the reproductive activity of *Culex* mosquitoes throughout the fall, winter, and spring in Ohio

**DOI:** 10.1186/s13071-023-05806-0

**Published:** 2023-05-31

**Authors:** Alden Siperstein, Laura W. Pomeroy, Sydney Robare, Lucas Sarko, Hannah Dehus, Taylor Lowmiller, Lydia Fyie, Megan E. Meuti

**Affiliations:** 1 Entomology at the Ohio State University, Room 216 Kottman Hall, 2001 Fyffe Dr., Columbus, OH 43210 USA; 2 Environmental Health Sciences, College of Public Health, Ohio State University, Columbus, USA; 3 Translational Data Analytics Institute, Ohio State University, Columbus, USA; 4 College of Public Health at the Ohio State University, Columbus, USA; 5 College of Nursing at the Ohio State University, 1577 Neil Avenue, Columbus, OH 43210 USA; 6 Clarke Global Environmental Products and Services, 2000 Kenton St, Columbus, OH 43205 USA

**Keywords:** *Culex pipiens*, *Culex erraticus*, *Culex restuans*, Diapause, Seasonality, Culvert, Overwintering, West Nile virus

## Abstract

**Background:**

*Culex* mosquitoes are the primary vectors of West Nile virus (WNV) across the USA. Understanding when these vectors are active indicates times when WNV transmission can occur. This study determined the proportion of female *Culex* mosquitoes that were in diapause during the fall and winter and when they terminated diapause and began blood feeding in the spring.

**Methods:**

Mosquitoes were collected from parks using various traps and/or aspirated from culverts in Franklin County, Ohio, from October to mid-May from 2019 to 2022. *Culex* mosquitoes were morphologically identified to species, and the ovaries of females were dissected to determine their diapause and parity statuses.

**Results:**

By early October 2021, roughly 95% of *Culex pipiens* collected in culverts were in diapause and 98% of *Cx. erraticus* were in diapause. Furthermore, gravid and blood-fed *Culex salinarius*, *Cx. pipiens*, and *Cx. restuans* were collected in late November in 2019 and 2021 in standard mosquito traps. In the winter of 2021, the proportions of non-diapausing *Culex* decreased within culverts. The last non-diapausing *Cx. erraticus* was collected in late December 2021 while the final non-diapausing *Cx. pipiens* was collected in mid-January 2022, both in culverts. Roughly 50% of *Cx. pipiens* terminated diapause by mid-March 2022, further supported by our collections of gravid females in late March in all 3 years of mosquito collection. In fact, male mosquitoes of *Cx. pipiens*, *Cx. restuans*, and *Cx. territans* were collected by the 1st week of May in 2022, indicating that multiple species of *Culex* produced a second generation that reached adulthood by this time.

**Conclusions:**

We collected blood-fed and gravid *Culex* females into late November in 2 of the 3 years of our collections, indicating that it might be possible for WNV transmission to occur in late fall in temperate climates like Ohio. The persistence of non-diapausing *Cx. pipiens* and *Cx. erraticus* throughout December has important implications for the winter survival of WNV vectors and our overall understanding of diapause. Finally, determining when *Culex* terminate diapause in the spring may allow us to optimize mosquito management programs and reduce the spread of WNV before it is transmitted to humans.

**Graphical Abstract:**

**Figure:**
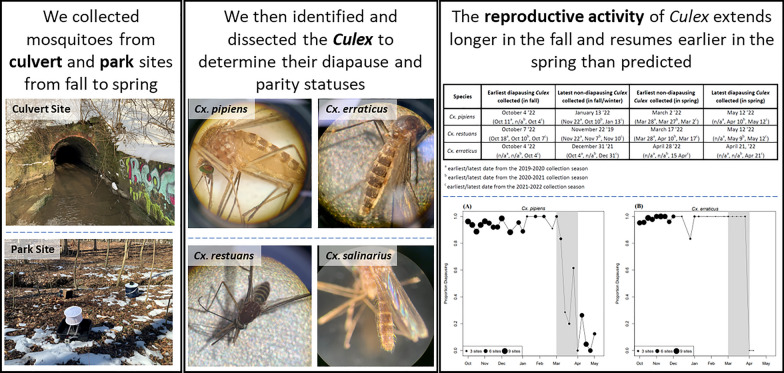

**Supplementary Information:**

The online version contains supplementary material available at 10.1186/s13071-023-05806-0.

## Background

West Nile virus (WNV) is the most common mosquito-borne pathogen in the continental USA [1]. Birds are the primary reservoirs of WNV, and transmission typically occurs after a mosquito consumes a blood meal from an infected avian host and then then bites another bird or human. In the US in 2022 there were 1035 confirmed human cases of WNV [2], and in the state of Ohio there were 7 confirmed cases, which unfortunately resulted in 1 death [2].

Human cases of WNV typically occur in mid-June to late October in Ohio, with peaks during mid-August [3]. As illness onset occurs anywhere from 2 to 14 days after a bite from an infected mosquito, residents of Ohio and other states at similar latitudes are at the greatest risk of becoming infected with WNV in early June through mid-October [3]. This seasonal distribution of WNV transmission is influenced by at least three important biological aspects of the mosquito vectors: competence for carrying and transmitting WNV, host preferences, and diapause.

Although many genera of mosquitoes have been found to transmit WNV and may serve as important bridge vectors (e.g. *Aedes vexans* and *Coquillettidia perturbans* [4–7]), *Culex* mosquitoes are the major vectors throughout the US [8–10]. During the spring and summer, *Cx. pipiens* and *Cx. restuans* are likely the primary vectors in the Northeastern US of WNV [9]. This is because both species are highly competent vectors of WNV [11, 12] and because both species primarily bite birds but will opportunistically bite mammals [13].

In addition to *Cx. pipiens* and *Cx. restuans*, three species of *Culex* are routinely collected in central Ohio: *Cx. salinarius*, *Cx. erraticus*, and *Cx. territans* [14]. The role of these remaining species in WNV transmission is currently unclear. Although *Cx. salinarius* may not play a large role in WNV transmission because of their lower vector competence [15], they may serve as an important bridge vector as they bite both birds and mammals [16]. Similarly, *Cx. erraticus* bite both birds and mammals [17], making them another potential bridge vector for WNV, especially as *Cx. erraticus* are frequently collected later in the year [18]. Previously, researchers collected WNV-infected *Cx. erraticus* from the field [19], even when overwintering [18]. However, these mosquitoes are often less abundant and therefore account for a lower proportion of WNV-infected mosquitoes relative to *Cx. pipiens*, *Cx. restuans*, and *Cx. salinarius* in the Northeastern US [9]. *Culex territans* primarily bite amphibians, and infrequently bite reptiles or mammals, and are therefore unlikely to transmit WNV to humans [17].

Diapause is an overwintering strategy common among adult *Culex* females in temperate areas that results in physiological and behavioral changes [20, 21]. Female larvae of *Cx. pipiens* subjected to short daylengths and low temperatures enter diapause as adults [22, 23]. Semi-field studies have found that 50% of *Cx. pipiens* initiate diapause around mid-September in Ames, Iowa (latitude 32.0°N; [24]) and at higher latitudes, diapause initiation occurs earlier with 50% of the population entering diapause around mid-July in Guelph, Ontario, Canada (latitude 43.4°N; [25]). One field study in Boston, Massachusetts (latitude 42.4°N; [26]) found that adult *Cx. pipiens* began entering diapause in mid-August and diapause incidence reached 50% by early September. While in diapause, adult female mosquitoes arrest ovarian development and store glycogen and lipids [27, 28]. Behaviorally, diapausing *Culex* females no longer seek vertebrate hosts [29], take blood meals [30], or lay eggs [31] but instead remain in secluded shelters (e.g. sewage tunnels/culverts, and caves; [32, 33]). However, not all diapausing *Culex* females survive harsh winter conditions, causing populations to reach their lowest abundance shortly after mosquitoes terminate diapause in the spring [33–36]. Therefore, diapause initiation corresponds to the cessation of WNV transmission in autumn while diapause termination allows WNV transmission to resume in the spring, especially because a small proportion of female *Cx. pipiens* and *Cx. erraticus* overwinter infected with WNV [18, 32, 37–39].

A better understanding of when *Culex* species initiate and terminate diapause will allow us to accurately predict seasonal cycles of WNV transmission and pinpoint when mosquito surveillance and control will be most effective. Therefore, our goal was to characterize how long *Culex* remain reproductively active throughout the fall and when they terminate diapause in the spring. Although previous semi-field studies have characterized how photoperiod and temperature induce diapause in larvae of *Cx. pipiens* [24, 25] and another study characterized the diapause incidence of female *Cx. pipiens* collected in a single overwintering site from August to mid-November [26], our study is unique in that it determined how long non-diapausing and reproductively active mosquitoes persist in the field across multiple sites. Additionally, by continuously collecting mosquitoes throughout the winter and spring, we were able to determine when *Cx. pipiens* terminate diapause and resume blood feeding over a 3-year period. We were also able to make strong inferences on when *Cx. erraticus* and *Cx. restuans* initiate and terminate their overwintering dormancies. Based on previous findings, we expected the majority of *Cx. pipiens* to cease host-seeking by October [24–26] and terminate diapause by mid-April [33, 39]. We found that most *Cx*. *pipiens* initiated diapause by October, yet a small proportion of *Cx. pipiens* along with many other *Culex* spp. remained reproductively active until later in the year. We also found that *Cx. pipiens* and *Cx. restuans* terminate diapause by mid-March.

## Methods

### Field sites and mosquito collection

Collections took place over a fall to spring period for 3 years. During the fall 2019 to spring 2020 collection season, gravid traps (upright-style CDC Gravid Trap Model 1712.11) baited with “stinky water” (a mixture of fermented grass clippings, milk protein, and yeast brewed outdoors for 4 days until November, when it then was brewed indoors) were placed at five field sites consisting of wooded parks and meadows around Franklin County, Ohio (40.0° N) and were collected roughly 24 h afterwards (Additional file 1: Fig. S1a). Traps were placed weekly from September 16th, 2019, until November 7th, 2019; then, on November 21st, December 12th, and once every 2 weeks from January 10th, 2020, to May 14th, 2020. Additionally, homemade resting box and floral bait traps were placed at these five sites from March 13, 2020, through May 14, 2020.

During the fall 2020 to spring 2021 collection season, gravid traps baited with stinky water (brew methods same as above), floral-baited BG Sentinel traps (Model 2883.11 baited with 1:1 linalool and nonanal), and resting box traps (Mosquito Resting Trap Model 2799 from BioQuip) were placed weekly at six lightly wooded parks from October 2nd, 2020, through November 12th, 2020. After four additional park sites were identified in November, mosquitoes were collected from a total of 10 lightly wooded parks once every 2 weeks from November 19th, 2020, through February 25th, 2021, using gravid, floral-baited BG Sentinel traps and resting box traps (Additional file 1: Fig. S1b). All three trap types were then placed weekly at all 10 parks from February 25th, 2021, through May 14th, 2021. Additionally, mosquitoes were aspirated from six culvert sites once every 2 weeks from April 2 through May 14th, 2021.

**Fig. 1:**
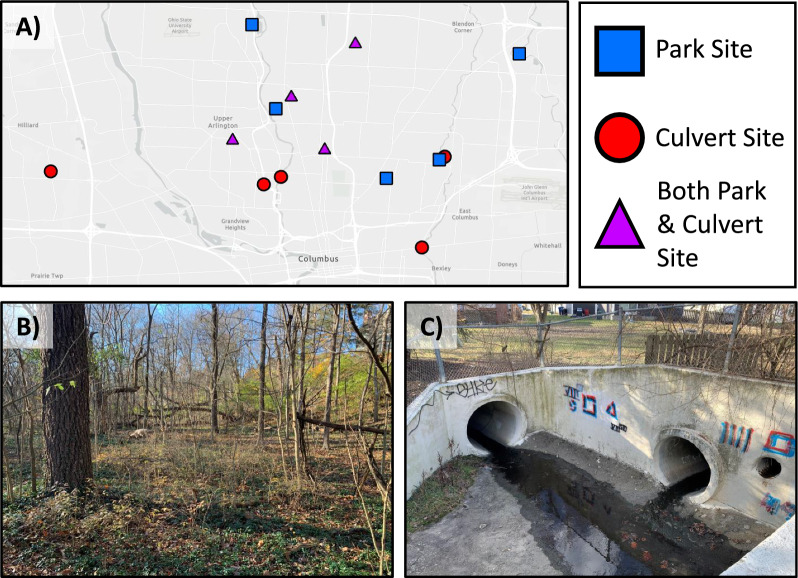
Distribution and examples of collection sites. **A** All 2021–2022 collection sites designed using ArcGIS pro [66]. Mosquito traps were placed in lightly wooded parks which are referred to as park sites (*n* = 5). Mosquitoes were aspirated from culvert sites (*n* = 5). Additionally, mosquitoes were collected from locations that contained both a park and culvert site (*n* = 4). **B** Example of a lightly wooded park site. **C** Example of a culvert site

During the fall 2021 to spring 2022 collection season, gravid traps baited with stinky water (brewed in an environmental chamber at 27 °C for 3 days) and BG Sentinel traps baited with CO_2_ and human scent lures of hexanoic acid were placed at nine park sites (Fig. 1a and b) and collected ~ 24 h afterwards. Traps were placed weekly from October 6th, 2021, through November 30th, 2021, and then once every 2 weeks until February 23rd, 2022. From March 2nd, 2022, until May 13th, 2022, gravid, BG Sentinel traps, and CDC light traps baited with CO_2_ and light were placed weekly at each park site. In addition to the nine park sites, mosquitoes were collected from nine culverts using mouth/mechanical aspirators on the same dates that mosquitoes were collected from park sites (Fig. 1a and c; Additional file 1: Fig. S1c).

### Species identification

Mosquito specimens were identified morphologically to species using keys in Craker and Collins [40]. The level of confidence in the morphological identification of mosquitoes was categorized as either “high” or “low.” PCR-based assays were used to confirm the morphological identifications of *Cx. pipiens*, *Cx. restuans*, *Cx. erraticus*, and *Cx. salinarius* (*n* = 36, 20, 7, and 10, respectively). In brief, genomic DNA was isolated from mosquito tissues using Phire Animal Tissue Direct or GeneJET Genomic DNA Purification kits. PCR assays were prepared using a universal reverse primer and three species-specific forward primers developed by Crabtree et al. [41] to distinguish *Cx. pipiens*, *Cx. restuans*, and *Cx. salinarius* while primers developed by Williams and Savage [42] were used to distinguish *Cx. erraticus*. PCRs were run using standard protocols for Phire Animal Tissue Direct PCR assays and DreamTaq Green Master Mix (2x; Additional file 1). Using this method, we determined that morphological identifications of 97.22% of *Cx. pipiens* (35/36), 100% of *Cx. restuans* (20/20), 100% of *Cx. erraticus* (7/7), and 50% of *Cx. salinarius* (5/10) were accurate. Additional PCR identifications were needed for samples identified to *Culex* or mosquitoes that were aspirated from the culverts and had initially been identified as *Cx. restuans* (Additional file 1).

### Diapause and parity status

Given the high number of samples we collected, we chose to assess the diapause and parity status of a subset of individuals. We first excluded female mosquitoes that were too damaged to be morphologically identified to species and those with missing thoraces or abdomens (*n* = 532 mosquitoes, leaving 3605 intact and morphologically identified *Culex* females). Then, we selected a maximum of 20 females that were collected from each site, collection date, and collection method. We preferentially selected female mosquitoes that had high confidence morphological identifications. If fewer than 20 females were captured with a particular collection method within a site/date, all females were dissected regardless of identification confidence. In total, we dissected 2480 female *Culex* mosquitoes (*n* = 425 for fall 2019–spring 2020; *n* = 133 for fall 2020–spring 2021; *n* = 1922 for fall 2021–spring 2022).

Ovaries were dissected from female mosquitoes under a stereomicroscope. To determine whether mosquitoes were parous, or had previously lain a batch of eggs, the tracheoles within to the ovaries were observed, where coiled tracheoles indicated that the mosquito was nulliparous and had not lain a batch of eggs, while uncoiled tracheoles indicated that the female was parous [39]. Mosquitoes that were parous with high certainty were classified as non-diapausing, regardless of egg follicle length. To determine the diapause status of nulliparous female mosquitoes, the lengths of the five largest primary egg follicles were measured using an inverted microscope (Nikon) at 200 × magnification. If the average length of the primary egg follicles was < 75 µm, the female mosquito was categorized as “diapausing” [43]. If the average egg follicle length was > 90 µm, female mosquitoes were classified as “non-diapausing” [43]. Those who were nulliparous and had egg follicle measurements between 75 µm and 90 µm were classified as “intermediate” [43].

### Data analysis

All analyses were conducted using R, version 4.1.2 [44], in RStudio [45]. The *maps* package [46] was used to generate maps of *Culex* abundance by species and collection site.

To determine when *Cx. pipiens* terminated diapause in the spring, we first grouped the *Cx. pipiens* samples collected from each culvert site and collection date. The proportions of *Cx. pipiens* in diapause in each culvert site was calculated as the ratio of diapausing *Cx. pipiens* divided by all *Cx. pipiens* that were dissected (e.g. diapausing, intermediate, and non-diapausing). Next, we plotted our diapause proportions from each site and each collection week and used a dose response curve to determine the time at which 50% of the *Cx. pipiens* terminated diapause (DTT or diapause termination time) and 95% CI using the effective dose function in the *drc* package in R [47].

Sample collection dates, mosquito abundance by genus, month, or collection method, species identifications for all mosquitoes collected including non-*Culex* genera abundances, dissection results, and coding for data analyses can all be found in the GitHub Repository in Availability of data and materials section.

## Results

### *Culex* species counts and seasonal distribution

Of the 4137 female *Culex* collected, 3605 female *Culex* were identified to species (Table 1; 122 male *Culex* collected; 83 male *Culex* were identified to species). During the fall 2019 to spring 2020 collection season, female and male *Culex* were collected almost exclusively from gravid traps (*n* = 845) and one female from a resting trap (*n* = 1), where the last female *Culex* were collected in late November and the first in mid-March (Table 2). The fall 2020 to spring 2021 collection season yielded far fewer *Culex* (Table 1); only 49 females were collected from gravid traps, 5 females from resting traps, 1 female from floral-baited BG Sentinel traps, and 98 from aspirating mosquitoes from culverts in the winter and spring. Notably, the last female *Culex* collected in park sites were collected in early November 2020 and the first in late March 2021 (Table 2). Most *Culex* were collected during the fall 2021 to spring 2022 collection season (Table 1); 664 female and male *Culex* were collected from park sites in traditional mosquito traps (Table 3) and were collected as late as mid-November and as early as mid-March. We collected 2596 female and male *Culex* from culverts during the 2021–2022 collection season (Table 3).

**Table 1:** Total counts of females from all *Culex* species that were collected across all three collection seasons

Species	2019–2020	2020–2021	2021–2022	Total
*Cx. erraticus*	2	0	807	809
*Cx. pipiens*	414	88	1621	2123
*Cx. restuans*	181	50	375	606
*Cx. salinarius*	3	2	13	18
*Cx. territans*	1	12	39	52
*Culex* not IDed to species	187	0	342	529

**Table 2:** Dates when diapausing or non-diapausing females of *Cx. pipiens*, *Cx. restuans* and *Cx. erraticus* were first collected in the fall and last collected in the spring during each collection season

Species	Earliest diapausing *Culex *collected (in fall)	Latest non-diapausing *Culex *collected (in fall/winter)	Earliest non-diapausing *Culex* collected (in spring)	Latest diapausing *Culex *collected (in spring)
*Cx. pipiens*	October 4 ′22 (Oct 11^a^, n/a^b^, Oct 4^c^)	January 13 ′22 (Nov 22^a^, Oct 10^b^, Jan 13^c^)	March 2 ′22 (Mar 28^a^, Mar 27^b^, Mar 2^c^)	May 12 ′22 (n/a^a^, Apr 10^b^, May 12^c^)
*Cx. restuans*	October 7 ′22 (Oct 18^a^, Oct 10^b^, Oct 7^c^)	November 22 ′19 (Nov 22^a^, Nov 7^b^, Nov 10^c^)	March 17 ′22 (Mar 28^a^, Apr 10^b^, Mar 17^c^)	May 12 ′22 (n/a^a^, May 9^b^, May 12^c^)
*Cx. erraticus*	October 4 '22 (n/a^a^, n/a^b^, Oct 4^c^)	December 31 ′21 (Oct 4^a^, n/a^b^, Dec 31^c^)	April 28 ′22 (n/a^a^, n/a^b^, Apr 15^c^)	April 21 '22 (n/a^a^, n/a^b^, Apr 21^c^)

^a^Earliest/latest date from the 2019–2020 collection season

^b^Earliest/latest date from the 2020–2021 collection season

^c^Earliest/latest date from the 2021–2022 collection season

**Table 3:** Number of female and male *Culex* mosquitoes by species that were collected using the four most effective collection techniques across each season

Fall 2019 – Spring 2020																
Species:	*Cx. pipiens*				*Cx. erraticus*				*Cx. restuans*				All* Culex*			
Traps:	*BG*	*CDC*	*Gravid*	*Culvert*	*BG*	*CDC*	*Gravid*	*Culvert*	*BG*	*CDC*	*Gravid*	*Culvert*	*BG*	*CDC*	*Gravid*	*Culvert*
September	N/A	N/A	9	N/A	N/A	N/A	0	N/A	N/A	N/A	4	N/A	N/A	N/A	29	N/A
October	N/A	N/A	81	N/A	N/A	N/A	1	N/A	N/A	N/A	7	N/A	N/A	N/A	116	N/A
November	N/A	N/A	6	N/A	N/A	N/A	0	N/A	N/A	N/A	3	N/A	N/A	N/A	6	N/A
December	N/A	N/A	0	N/A	N/A	N/A	0	N/A	N/A	N/A	0	N/A	N/A	N/A	0	N/A
January	N/A	N/A	0	N/A	N/A	N/A	0	N/A	N/A	N/A	0	N/A	N/A	N/A	0	N/A
February	N/A	N/A	0	N/A	N/A	N/A	0	N/A	N/A	N/A	0	N/A	N/A	N/A	0	N/A
March	N/A	N/A	40	N/A	N/A	N/A	0	N/A	N/A	N/A	25	N/A	N/A	N/A	90	N/A
April	N/A	N/A	70	N/A	N/A	N/A	0	N/A	N/A	N/A	27	N/A	N/A	N/A	143	N/A
May	N/A	N/A	244	N/A	N/A	N/A	1	N/A	N/A	N/A	150	N/A	N/A	N/A	461	N/A

Fall 2020 – Spring 2021																
Species:	*Cx. pipiens*				*Cx. erraticus*				*Cx. restuans*				All* Culex*			
Traps:	*BG*	*CDC*	*Gravid*	*Culvert*	*BG*	*CDC*	*Gravid*	*Culvert*	*BG*	*CDC*	*Gravid*	*Culvert*	*BG*	*CDC*	*Gravid*	*Culvert*
October	N/A	N/A	4	N/A	N/A	N/A	0	N/A	N/A	N/A	4	N/A	N/A	N/A	8	N/A
November	N/A	N/A	1	N/A	N/A	N/A	0	N/A	N/A	N/A	2	N/A	N/A	N/A	3	N/A
December	N/A	N/A	0	N/A	N/A	N/A	0	N/A	N/A	N/A	0	N/A	N/A	N/A	0	N/A
January	N/A	N/A	0	12	N/A	N/A	0	0	N/A	N/A	0	0	N/A	N/A	0	14
February	N/A	N/A	0	N/A	N/A	N/A	0	N/A	N/A	N/A	0	N/A	N/A	N/A	0	N/A
March	N/A	N/A	1	N/A	N/A	N/A	0	N/A	N/A	N/A	0	N/A	N/A	N/A	1	N/A
April	N/A	N/A	15	45	N/A	N/A	0	0	N/A	N/A	6	20	N/A	N/A	21	69
May	N/A	N/A	6	2	N/A	N/A	0	0	N/A	N/A	8	7	N/A	N/A	16	15

Fall 2021 – Spring 2022																
Species:	*Cx. pipiens*				*Cx. erraticus*				*Cx. restuans*				All* Culex*			
Traps:	*BG*	*CDC*	*Gravid*	*Culvert*	*BG*	*CDC*	*Gravid*	*Culvert*	*BG*	*CDC*	*Gravid*	*Culvert*	*BG*	*CDC*	*Gravid*	*Culvert*
October	4	N/A	17	560	1	N/A	2	481	0	N/A	8	6	18	N/A	38	1189
November	2	N/A	2	476	0	N/A	0	247	0	N/A	0	3	6	N/A	5	755
December	0	N/A	0	157	0	N/A	0	33	0	N/A	0	0	0	N/A	0	202
January	0	N/A	0	70	0	N/A	0	16	0	N/A	0	0	0	N/A	0	88
February	0	N/A	0	39	0	N/A	0	8	0	N/A	0	0	0	N/A	0	47
March	0	0	3	53	0	0	0	14	1	0	2	2	1	0	5	74
April	3	1	11	59	0	0	0	4	0	0	3	27	5	1	16	103
May	3	4	120	51	0	0	0	1	4	0	264	71	8	4	556	138

The duration of female reproductive activity varied depending on the collection year. Non-diapausing *Cx. pipiens* were collected in park sites as late as November in each collection season and as early in the spring as mid- to late March, depending on the collection season (Table 2). *Culex pipiens* were collected from culverts for the entire duration of the 2021 to 2022 collection season (Table 3).

Aspirating mosquitoes from the culverts yielded far higher collection numbers of *Cx. erraticus* than the BG Sentinel and gravid traps placed at the park sites in the fall 2021–spring 2022 collection season (Table 3). Non-diapausing *Cx. erraticus* were collected in park sites as late as early to mid-October from the 2019 and 2021 seasons and as early in the spring as early May in 2020 (Table 2). Furthermore, we only collected one non-diapausing female *Cx. erraticus* from a culvert in late April and one additional non-diapausing female *Cx. erraticus* in May (Additional file 1: Table S1).

**Fig. 2:**
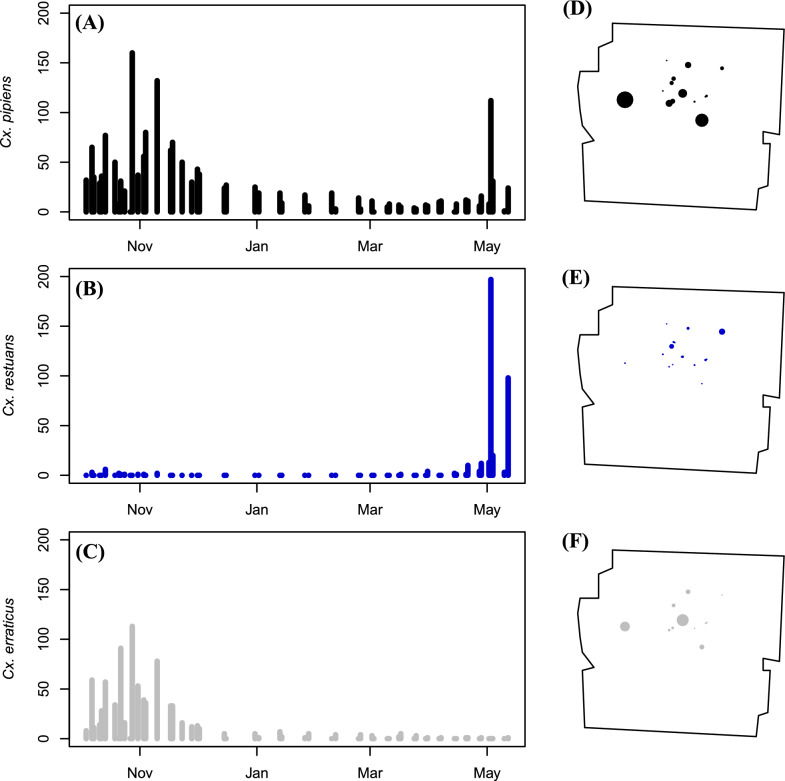
Counts of *Cx. pipiens* (**A**), *Cx. restuans* (**B**), and *Cx. erraticus* (**C**) collected across time and relative abundance of *Cx. pipiens* (**D**), *Cx. restuans* (**E**), and *Cx. erraticus* (**F**) at various collection sites in Franklin County, Ohio, during the 2021 to 2022 collection season. For panels **D**–**F** the size of the point at each site is proportional to the number of mosquitoes collected at that site

**Fig. 3:**
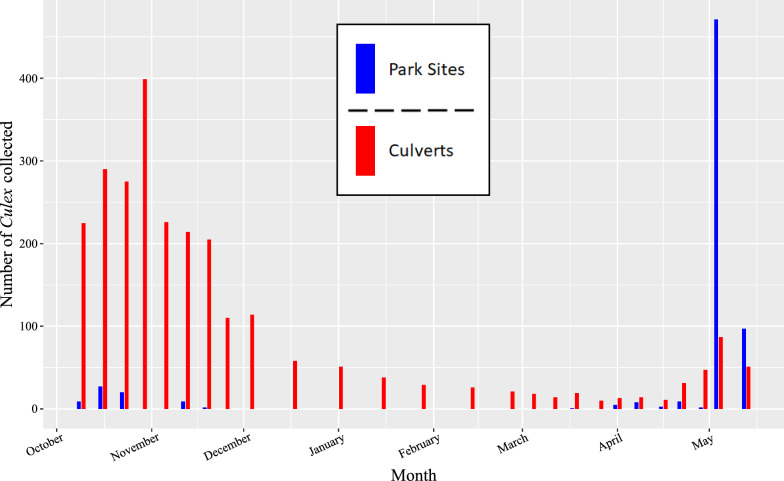
Counts of all *Culex* collected from traditional mosquito traps placed at park sites (blue) and counts of mosquitoes aspirated from culvert sites (red) during the 2021–2022 collection season. Mosquitoes were initially collected from park sites using gravid trap and BG Sentinel traps from October–February; starting in March, CDC light traps were also used weekly. Mosquitoes were collected from culverts using aspirators throughout the collection season

*Culex pipiens* and *Cx. erraticus* were commonly found in the same culvert sites throughout the fall 2021 to spring 2022 collection season yet differed in population abundance across sites (Fig. 2d and f). *Culex pipiens* were more abundant than *Cx. erraticus* at all collection sites except one, a train underpass, where 170 *Cx. pipiens* and 244 *Cx. erraticus* were collected. In fact, more *Culex* mosquitoes were collected from culverts than from traps in park sites throughout the 2021–2022 collection season until the first week of May (Fig. 3).

Like other species, the seasonal abundance and distribution of *Cx. restuans* varied among collection sites (Fig. 2c and e). Non-diapausing *Cx. restuans* were collected in park sites as late as mid-November during the 2019 and 2021 seasons and as early in the spring as mid-March to mid-April (Table 2). *Culex restuans* could not be collected year-round from culverts, and the few females collected from culverts in the fall of 2021 were non-diapausing (*n* = 9 collected; 5 dissected with 100% non-diapause; Table 3). *Culex restuans* were less abundant than *Cx. pipiens* or *Cx. erraticus* throughout the 2021–2022 collection season until the 1st week of May (Fig. 2ac).

*Culex territans* and *Cx. salinarius* were less abundant than the other *Culex* species (Table 1). No non-diapausing *Cx. territans* were collected from park sites during the fall or winter in any collection season, yet diapausing females were collected in BG Sentinel traps as late as 21 October in fall 2021 and diapausing females of *Cx. territans* were collected throughout the fall and winter 2021 within culverts. The only months when female *Cx. territans* were not collected from culverts were February and March. Gravid *Cx. territans* were collected in culverts in mid-April. *Culex salinarius* were collected from mid-October to mid-November, depending on the collection season. In fall 2021, only 1 female of *Cx. salinarius* was collected within a culvert while 12 females were collected from BG Sentinel traps. Across all collection seasons only one female of *Cx. salinarius* was ever collected in the spring. This sample was collected on May 2, 2020, from a gravid trap.

### *Culex* reproductive activity

**Fig. 4:**
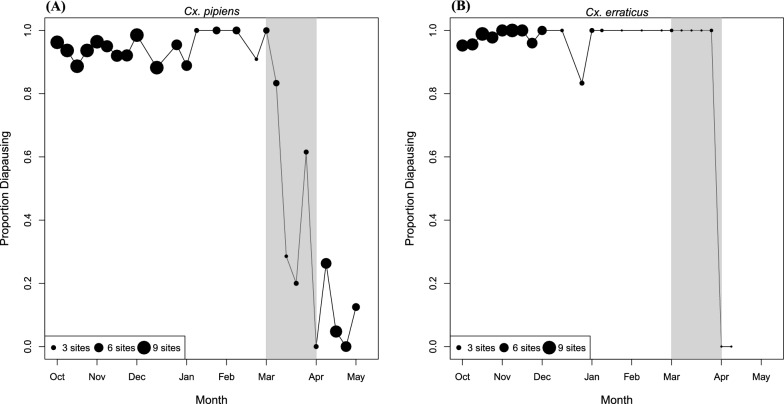
The proportions of diapausing *Cx. pipiens* (**A**) and *Cx. erraticus* (**B**) collected from culverts in fall 2021 through spring 2022. The size of each point is proportional to the number of culverts where *Culex* were collected. Gray shading highlights dates between March 1, 2022, and April 21, 2022, when there was a rapid decrease in the proportion of mosquitoes in diapause. Although all nine culverts were sampled during each collection event, *Cx. pipiens* and/or *Cx. erraticus* were not necessarily collected from every culvert on every collection date

*Culex pipiens* populations exhibited low rates of reproductive activity throughout the fall that ceased in the early winter. *Culex pipiens* then resumed reproductive activity in early spring. Non-diapausing *Cx. pipiens* were collected in November during each collection season (Table 2). Non-diapausing *Cx. pipiens* were also found within culverts in October, November, and December 2021 and January 2022. In fact, roughly 6% of *Cx. pipiens* collected from culverts in October 2021 that we dissected were in a non-diapause or intermediate state (Fig. 4a). The proportion of non-diapausing *Cx. pipiens* within culverts decreased during November and December 2021, such that by the 2nd week of January 2022, 100% of *Cx. pipiens* were in a diapause state (Fig. 4a). Interestingly, the non-diapausing *Cx. pipiens* from December showed no higher rates of *Cx. pipiens* form *molestus* introgression than diapausing *Cx. pipiens* in December (Additional file 1: Supplemental Materials and Methods and Supplemental Results). Diapause incidence reached 100% by the 2nd week of January in 2022 and remained 100% until the first week of March 2022 (Fig. 4), at which point a single non-diapausing female of *Cx. pipiens* was collected within a culvert (Table 2). Our analyses indicate 50% of the *Cx. pipiens* within culverts terminated diapause by March 24, 2022 (dose-response curve; SE = 4.83 days; df = 151; Additional file 1: Fig. S2). The proportion of diapausing *Cx. pipiens* in culverts remained consistently low throughout April and May (Fig. 4a; ranged from 0 to 26.3% between weeks). Additionally, the proportion of *Cx. pipiens* in diapause varied among individual culverts (Additional file 1: Fig. S3).

The proportion of diapausing *Cx. erraticus* in the fall was higher than for *Cx. pipiens*, where only 1.2% of female *Cx. erraticus* collected from the culverts in October 2021 were in a non-diapause or intermediate state (Fig. 4b). Additionally, the proportion of diapausing *Cx. erraticus* within culverts varied (Additional file 1: Fig. S3b). During November 2021, only one non-diapausing female was collected from a park trap, and the last non-diapausing *Cx. erraticus* was collected from a culvert on December 31, 2021 (parous sample with average egg follicle length = 66 µm; Table 2). As collection numbers substantially decreased throughout the winter and did not rebound in early spring, it is unclear when females of *Cx. erraticus* terminated diapause and became reproductively active (Fig. 4b). However, two non-diapausing *Cx. erraticus* were collected from culverts in the spring of 2022; the first was collected on April 28th (gravid female) and the second on May 12th (nulliparous female with an average egg follicle length of 105 µm).

Non-diapausing females of *Cx. restuans* were collected until mid to late November during the 2019 and 2021 collection seasons (Table 2). The first non-diapausing females of *Cx. restuans* were collected in mid-March to early April depending on the collection season (Table 2). In fact, the first *Cx. restuans* collected in spring 2022 was a gravid female who was collected from a gravid trap (Table 3). The abundance of *Cx. restuans* substantially increased over the spring (Fig. 2b). By the 1st week of May 2022, 196 non-diapausing females of *Cx. restuans* were collected in gravid traps (averaging 21.78 per site) and 32 non-diapausing female *Cx. restuans* were collected from culverts (averaging 3.55 per site).

The presence of blood-fed and gravid females of *Cx. pipiens* and *Cx. restuans* further provide evidence of reproductive activity. In the fall, blood-fed females of both species were collected until November 10th in 2021 (Table 4). The last gravid *Cx. restuans* was found on November 22nd in 2019 whereas the last gravid *Cx. pipiens* was collected on December 31st in 2021 (Table 4). In the spring, the first gravid *Cx. restuans* was collected on March 17th in 2022 whereas the first gravid *Cx. pipiens* was collected by late March for all three collection seasons (Table 4).

**Table 4:** Dates when gravid or blood-fed females of *Cx. pipiens* and *Cx. restuans* were last collected in the fall/winter and first collected in the spring during each collection season

Species	Last blood-fed female collected (in fall)	First blood-fed female collected (in spring)	Last gravid female collected (in fall/winter)	First gravid female collected (in spring)
*Cx. pipiens*	November 10 ′21 (n/a^a^, n/a^b^, Nov 10^c^)	March 25 ′22 (n/a^a^, Apr 2^b^, Mar 25^c^)	December 31 ′21 (Nov 22^a^, Nov 7^b^, Dec 31^c^)	March 27 ′21 (Mar 28^a^, Mar 27^b^, Mar 31^c^)
*Cx. restuans*	November 10′21 (n/a^a^, n/a^b^, Nov 10^c^)	April 15 ′22 (n/a^a^, n/a^b^, Apr 15^c^)	November 22 ′19 (Nov 22^a^, Nov 7^b^, Oct 22^c^)	March 17 ′22 (Mar 28^a^, Apr 10^b^, Mar 17^c^)

^a^Earliest/latest date from the 2019–2020 collection season

^b^Earliest/latest date from the 2020–2021 collection season

^c^Earliest/latest date from the 2021–2022 collection season

As male *Culex* mosquitoes do not overwinter, the presence of males in early spring also indicates when the first generation of mosquitoes produced by post-diapause *Culex* females reaches reproductive maturity. Notably, no male *Culex* were found in any culvert or park sites after early December 2021, indicating that all males had perished by this time. However, by the 1st week of May 2022, four adult males of *Cx. pipiens* were collected from two sites (2 collected from a gravid trap; 2 collected from a culvert). Similarly, three males of *Cx. restuans* and two males of *Cx. territans* were collected in a culvert site. Subsequently, by the 2nd week of May, we found 4 males of *Cx. pipiens* from 4 sites (1 in a CDC light trap; 1 from three different culverts), 10 males of *Cx. restuans* from 3 separate culvert sites (*n* = 6, 3, and 1 for each culvert site), and 1 male *Cx. territans* from a culvert. Collectively, this indicates that by early May, multiple species of *Culex* had completed at least one full life cycle.

## Discussion

By continuously collecting mosquitoes from multiple field sites over a 3-year period, we were able to uncover several interesting results that have important implications for WNV transmission. First, several species of *Culex*, including *Cx. pipiens, Cx. restuans*, and *Cx. salinarius*, are reproductively active into late November, although this varied slightly among collection years. Despite differences in collection methods, we consistently collected reproductively active *Cx. pipiens* in gravid traps until November. This demonstrates that reproductively active mosquitoes persist through the fall. Second, both *Cx. pipiens* and *Cx. erraticus* overwinter within culverts, and a low proportion of non-diapausing female *Cx. pipiens* persist until mid-January. Third, the proportion of diapausing *Cx. pipiens* dramatically decreased throughout the month of March 2022, and gravid *Cx. pipiens* were collected by late March in gravid traps across all three collection seasons, indicating that *Cx. pipiens* can consistently be collected in early spring using a common surveillance trap.

Previous studies have also reported low incidences of non-diapausing *Cx. pipiens* in late fall and early winter [39, 48, 49]. A closely related and interfertile subspecies of *Cx. pipiens*, *Cx. pipiens* form *molestus*, hereafter *Cx. p. molestus*, is commonly found underground and does not enter diapause [50–53]. We were curious whether the non-diapausing females of *Cx. pipiens* that we had collected in December and early January belonged to this sub-species and/or showed higher rates of *Cx. p. molestus* ancestry, especially as other investigators have reported this phenomenon [49]. However, we found no evidence of higher rates of *Cx. p. molestus* introgression in our non-diapausing samples (Additional file 1: Supplemental Materials & Methods and results; [54]). Therefore, we propose three possible explanations for the presence of non-diapausing *Cx. pipiens* within culverts in December and January. First, these *Cx. pipiens* females that had not entered diapause may simply be holdouts that emerged earlier in the year. As they had not initiated diapause and lacked resources to survive the winter, this would explain why these females were not collected after late January. Notably, other studies that collected overwintering *Cx. pipiens* [39, 48, 49] report a similar trend and reached this same conclusion. A second possibility is that a small proportion of local *Cx. pipiens* that emerge in September or October may not enter diapause and similarly eventually perish during hard winters. Notably, Batz et al. [55] report that a low proportion of *Aedes albopictus* do not enter diapause under strong diapause-inducing conditions, and we also observed this in our laboratory colony of *Cx. pipiens* [56]. This small proportion of the *Cx. pipiens* that averts diapause may do so as a bet-hedging mechanism, enabling these females to avoid the reproductive costs of entering diapause and increase the mean geometric fitness of the population in the event of a mild winter [57–59]. Finally, the small proportion of the population that averts diapause could be caused by human-mediated changes to the urban environment such as the urban heat island effect (Fyie et al. *accepted*) and artificial light at night [60]. Notably, none of these possibilities is mutually exclusive, and all three could explain why we collected non-diapausing *Cx. pipiens* in culverts through mid-January.

Our collection of male *Cx. restuans*, *Cx. territans*, and *Cx. pipiens* by the 1st week of May 2022 indicates that at least three species of *Culex* had terminated diapause and completed at least one generation before most mosquito districts began their adult surveillance efforts (week 20; FCPH). Notably, Ciota et al. [33] uncovered annual variation when *Cx. pipiens* terminated diapause in New York, and therefore surveillance over multiple years may be necessary to determine which environmental factors influence diapause termination in multiple *Culex* spp. so that we can accurately predict when cycles of WNV transmission are likely to reinitiate. Although previous studies have demonstrated that the overwintering survivorship of *Cx. pipiens* can vary, the literature consistently demonstrates that populations of *Cx. pipiens* are lowest in early spring [33, 48, 61]. Additionally, our data showcase how rapidly mosquito populations can increase during the spring and are consistent with Helbing et al.'s [62] finding that populations of *Cx. pipiens* and especially *Cx. restuans* rapidly increase throughout May in Lucas County, Ohio. Therefore, identifying when *Culex* mosquitoes terminate diapause and emerge from their hibernacula in the spring can be applied to improve integrated pest management. This control method would target mosquito populations when they are at their lowest, hopefully leading to reduced WNV transmission throughout the year.

We found several interesting differences in the abundance and species composition of *Culex* mosquitoes within culverts and above-ground sites throughout the year in Franklin County, Ohio. Two vectors of WNV overwinter within culverts, *Cx. pipiens* and *Cx. erraticus*, whereas *Cx. restuans* and *Cx. salinarius* do not. Although Nasci et al. [32] collected low numbers of *Cx. restuans* from various humanmade overwintering sites in Queens, NY, we did not collect any diapausing or non-diapausing *Cx. restuans* within culverts during winter. Therefore, the mystery of where *Cx. restuans* overwinter in the field remains. *Culex salinarius* were almost exclusively collected from BG Sentinel traps in the fall, and only one female was collected in a culvert during the winter. A previous study indicates that *Cx. salinarius* can be collected from animal burrows and other natural habitats as opposed to humanmade sites [63]. It is also unclear whether *Cx. salinarius* enter a true reproductive diapause or whether they stop host-seeking and enter an overwintering quiescence in direct response to low temperatures [64, 65]. Unfortunately, our results are similarly unable to address this question. In the future, however, better characterizing the diapause response of multiple *Culex* species and identifying where they overwinter will allow us to better understand the relative contribution of each species to the seasonal dynamics of WNV transmission.

Unlike traditional mosquito traps that collect host-seeking and/or gravid mosquitoes, aspirating mosquitoes from culverts allowed us to collect *Cx. pipiens*, *Cx. erraticus*, and, to a lesser extent, *Cx. territans* year-round. Using these collection methods, future studies can determine which environmental cues are most relevant for diapause termination and specifically whether variations in microclimate between culverts influence when mosquitoes exit from hibernacula. Moreover, future studies can assess the proportion of overwintering *Cx. pipiens* or *Cx. erraticus* that are infected with WNV and to what extent diapausing mosquitoes serve as reservoir for this and other arboviruses [18, 32, 37–39]. Future studies that collect mosquitoes from both rural and urban areas at similar latitudes will allow researchers to determine the impact of human-mediated changes to the environment on the timing and duration of diapause in *Culex* and predict how urbanization alters the seasonal dynamics of WNV transmission.

## Conclusion

The overall objective of this study was to determine when different species of *Culex* were reproductively active. We specifically focused on when *Cx. pipiens*, the region’s primary vector of WNV, ceased host-seeking and reproducing in the fall and terminated its diapause in the spring. In doing so, we have found that many different *Culex* species and other mosquitoes remain reproductively active and can be collected with traditional mosquito traps (e.g. gravid and BG Sentinel traps) as late as November in the fall and as early as late March in the spring in Franklin County, Ohio. However, these traps failed to collect the mosquitoes through late November and mid-March, indicating that *Culex* mosquitoes are largely inactive and are likely in diapause during this time. In contrast, three species, *Cx. pipiens*, *Cx. erraticus*, and *Cx. territans*, could be collected only from culverts throughout the fall, winter, and early spring. By dissecting and examining the ovaries of female mosquitoes collected throughout the fall, winter, and spring, we determined that most females of *Cx. pipiens* terminated diapause by mid-March. Our findings and those of other studies indicate that *Culex* populations are lowest early in spring [33, 35]. Therefore, our results suggest that targeted pesticide applications in early spring could potentially provide a novel opportunity to effectively control mosquitoes before they are able to transmit deadly pathogens to birds, humans, and other animals.

## Supplementary Information

**Additional file 1: **Methods. **Figure S1.** The location of collection sites used in each collection season. **A** Fall 2019–spring 2020; **B** fall 2020–spring 2021; **C** fall 2021–spring 2022. **Figure S2.** Dose-response curve fitted onto the proportion of *Culex pipiens* that were in diapause collected from each culvert site and each week from the 2021–2022 collection season. The gray line indicates the 95% confidence interval. Our analyses indicate that 50% of *Cx. pipiens* had terminated diapause by March 24 in 2022. **Figure S3.** The proportion of *Culex pipiens* **A** and *Cx. erraticus* **B** in diapause over time varied across nine different culvert sites. Lines plotted are smoothing splines with three degrees of freedom. Each of the nine culverts are plotted in a different color. **Table S1**. The number of female *Culex *mosquitoes by diapause status that were collected each month during each collection season. Note that “*D*” refers to diapausing, “*ND*” refers to non-diapausing, “*Int.*” refers to intermediate, and “*Undis.*” refers to undissected mosquito samples.

## Data Availability

Please visit our GitHub Repository for additional information: https://github.com/AldenDSiperstein/Culex-Surveillance-P-V-Data-Availability. Any additional information is available from the corresponding author upon request.

## Abbreviations

WNV: West Nile virus
CDC: Center for Disease Control
BG: BioGents^®^
FCPH: Franklin county public health
