# Supplementary material for: Characterizing seasonal changes in the reproductive activity of Culex mosquitoes throughout the fall, winter, and spring in Ohio
Source: Parasit Vectors. 2023 May 31;16:173. doi: 10.1186/s13071-023-05806-0 (PMC10230737; doi:10.1186/s13071-023-05806-0)
Supplement: Supplementary file 1 — Additional file 1: Methods. Figure S1. The location of collection sites used in each collection season. A Fall 2019–spring 2020; B fall 2020–spring 2021; C fall 2021–spring 2022. Figure S2. Dose-response curve fitted onto the proportion of Culex pipiens that were in diapause collected from each culvert site and each week from the 2021–2022 collection season. The gray line indicates the 95% confidence interval. Our analyses indicate that 50% of Cx. pipiens had terminated diapause by March 24 in 2022. Figure S3. The proportion of Culex pipiens A and Cx. erraticus B in diapause over time varied across nine different culvert sites. Lines plotted are smoothing splines with three degrees of freedom. Each of the nine culverts are plotted in a different color. Table S1. The number of female Culex mosquitoes by diapause status that were collected each month during each collection season. Note that “D” refers to diapausing, “ND” refers to non-diapausing, “Int.” refers to intermediate, and “Undis.” refers to undissected mosquito samples. [file 13071_2023_5806_MOESM1_ESM.docx]

**Additional File 1:** **Supplementary Information**

**Additional file 1 & Methods:**

**PCR Protocols for determining *Culex* species:**

We ran PCRs on dissected female *Culex* that we only identified to the *Culex* genus. As many of the samples that were aspirated from the culvert sites and initially identified as *Cx. restuans* had not amplified with the Crabtree et al. [41] primers, we also ran PCRs on these dissected samples to determine if they were *Cx. erraticus* instead. Based on body size, coloration, and time of year, we pooled mosquitoes (n = 397) or tested them individually (n = 167). The pooled samples contained 5 mosquitoes/reaction maximum and were pooled only if collected from the same site and day. The gDNA of these samples then were divided equally to 1 µL per PCR tube using the Phire Animal Tissue Direct with a forward and reverse primer set for identifying *Cx. erraticus* developed by Williams & Savage [42] and ran using standard Phire Animal Tissue Direct Protocol.

Phire Animal Tissue Direct PCR assays contained 10 µl of Phire Animal Tissue PCR Buffer, 0.4 µl of Phire Hot Start 2, 500 nmol of each primer, molecular grade H_2_O, and 1 µl of gDNA (total reaction volume = 20 µl), and the PCR conditions were: 98˚C, 5 min followed by 40 cycles of 98˚C for 5 sec, 54.4˚C for 5 sec and 72˚C for 20 sec, and 72˚C for 1 min. The DreamTaq Green PCR Master Mix (2x) PCR assays contained 10 µL of DreamTaq Green Master Mix (2x), 500 nmol of each primer, molecular grade H_2_O, and 2 µL of gDNA (total reaction volume = 20 µL), and the reaction conditions were: 95˚C, 3 min, followed by 39 cycles of 95˚C for 30 sec, 46.5˚C for 30 sec, 72˚C for 30 sec and 72˚C for 5 min. Following amplification, PCR products were run on a 1% agarose gel.

**Determining *Cx. pipiens* form *molestus* Ancestry:**

To determine if *Cx. pipiens* collected in December and January that were gravid or non-diapausing showed greater introgression with Cx. p. molestus, we ran PCRs for two groups: 8 non-diapausing *Cx. pipiens* collected from culverts in December and January (n = 5 gravid; n = 3 with EFL > 90 μm) and 8 diapausing samples that were collected from culverts during the same timeframe (nulliparous & EFL < 75 μm). gDNA was extracted from one leg of each mosquito using the Phire Animal Tissue Direct 4.1.2 Dilution Protocol (ThermoFisher Scientific). PCRs were run with Phire Animal Tissue Direct PCR assays containing 10 µl of Phire Animal Tissue PCR Buffer, 0.4 µl of Phire Hot Start 2, 500 nmol of each primer (n = 3; primers as from Bahnck & Fonseca [65] CQ11 rapid assay), molecular grade H_2_O, and 1 µl of gDNA (total reaction volume = 20 µl). The PCR conditions were as follows: 98˚C, 5 min followed by 40 cycles of 98˚C for 5 sec, 54˚C for 5 sec and 72˚C for 20 sec, and 72˚C for 1 min. A Fisher's exact test was used to determine if the non-diapausing group had higher rates of *Cx. p. molestus* ancestry than the diapausing group (α = 0.05).

**Additional file Results**:

**Non-diapausing *Cx. pipiens* collected in the winter do not have higher rates of *Cx. p. molestus* ancestry:**

There was no significant difference in *Cx. p*. *molestus* ancestry between diapausing and non-diapausing *Cx. pipiens* (Fisher’s exact test, p = 0.5692, OR = 3.838968, 95% CI = 0.2272195-250.8760421). Notably, 1/8 non-diapausing females showed signs of introgression with *Cx. p. molestus* while 3/8 diapausing females did.

**Additional file Reference**:

66. Bahnck CM, Fonseca DM. Rapid assay to identify the two genetic forms of *Culex* (*Culex*) *pipiens* L. (*Diptera: Culicidae*) and hybrid populations. Am J Trop Med Hyg. 2006;75. <https://doi.org/10.4269/ajtmh.2006.75.2.0750251>.

**Additional file Figure Captions:**

**Figure S1**: The location of collection sites used in each collection season. (A) Fall 2019 – Spring 2020 (5 park sites); (B) Fall 2020 - Spring 2021 (10 park sites and 6 culvert sites); and (C) Fall 2021 – Spring 2022 (5 park sites, 5 culvert sites, and 4 sites that contained both a park and culvert).

**Figure S2**. Dose-Response curve fitted onto the proportion of *Cx. pipiens* that were in diapause collected from each culvert site and each week from the 2021 – 2022 collection season. The gray line indicates the 95% confidence interval. Our analyses indicate that 50% of *Cx. pipiens* had terminated diapause by March 24^th^ in 2022 (SE = 4.83 days; df = 151).

**Figure S3**. The proportion of *Cx. pipiens* (A) and *Cx. erraticus* (B) in diapause over time varied across nine different culvert sites. Lines plotted are smoothing splines with three degrees of freedom. Each of the nine culverts are plotted in a different color.

**Additional file Table Caption:**

**Table S1**: The number of female *Culex* mosquitoes by diapause status that were collected each month during each collection season. Note that “*D*” refers to diapausing, “*ND*” refers to non-diapausing, “*Int.*” refers to intermediate, and “*Undis.*” refers to undissected mosquito samples.
